# Electromagnetic and Thermal Simulations of Human Neurons for SAR Applications

**DOI:** 10.4236/jbise.2016.99039

**Published:** 2016-08-12

**Authors:** Felipe Perez, Gilbert Millholland, Seshasai Vamsi Krishna Peddinti, Ashok Kumar Thella, James Rizkalla, Paul Salama, Maher Rizkalla, Jorge Morisaki, Maher E. Rizkalla

**Affiliations:** 1 Department of Medicine, Indiana University School of Medicine, Indianapolis, IN, USA; 2 Department of Electrical and Computer Engineering (ECE), Indiana University Purdue University Indianapolis (IUPUI), Indianapolis, IN, USA; 3 Indiana University School of Medicine, Indiana University Purdue University Indianapolis (IUPUI), Indianapolis, IN, USA; 4 Department of Bioengineering, University of Illinois at Chicago, Chicago, IL, USA; 5 Integrated Nanosystems Development Institute (INDI), Indiana University Purdue University Indianapolis (IUPUI), Indianapolis, IN, USA

**Keywords:** EM (Electromagnetic), SAR (Specific Absorption Rate), COMSOL, HFSS, HN (Human Neuron)

## Abstract

The impact of the electromagnetic waves (EM) on human neurons (HN) has been under investigation for decades, in efforts to understand the impact of cell phones (radiation) on human health, or radiation absorption by HN for medical diagnosis and treatment. Research issues including the wave frequency, power intensity, reflections and scattering, and penetration depths are of important considerations to be incorporated into the research study. In this study, computer simulation for the EM exposure to HN was studied for the purpose of determining the upper limits of the electric and magnetic field intensities, power consumption, reflections and transmissions, and the change in temperature resulting from the power absorption by human neurons. Both high frequency structural simulators (HFSS) from ANSYS software, and COMSOL multi-physics were used for the simulation of the EM transmissions and reflections, and the temperature profile within the cells, respectively. For the temperature profile estimation, the study considers an electrical source of 0.5 watt input power, 64 MHz. The EM simulation was looking into the uniformity of the fields within the sample cells. The size of the waveguide was set to be appropriate for a small animal model to be conducted in the future. The incident power was fully transmitted throughout the waveguide, and less than 1% reflections were observed from the simulation. The minimum reflected power near the sample under investigation was found to be with negligible reflected field strengths. The temperature profile resulting from the COMSOL simulation was found to be near 0.25 m°K, indicating no change in temperature on the neuro cells under the EM exposure. The paper details the simulation results for the EM response determined by HFSS, and temperature profile simulated by COMSOL.

## 1. Introduction

For decades, researchers have investigated the effects of EM radiation on biological systems and human health, including the effects of cell phones and power lines. EM radiation may have negative effects on genetic material (DNA) by UV and X-rays [1]. The frequency signals sometimes are magnified by cell phone companies in response to the traffic flow and contention issues, resulting in higher power consumption within the human bodies, and this even raises more concern on the impact on the center nervous system of the human body. High level of EM energy radiation may also impact the SAR that may raise safety issues. However, not every biological effect initiated by EM energy radiation is a health hazard. In fact, worldwide development of bioelectromagnetic medicine clearly indicates that properly chosen EMF/EM/MF and electric current may be beneficial in treatment of various disease and injuries, even when all other known medical treatment failed [2]. The exposure of human cells to SAR values between 5 and 25 W/Kg may have effect on transcription and cell structure [3]. The maximum limit of accepted power absorption to partial body exposure for uncontrolled environments is established by US and Europe to be 1.6 W/kg, and 2 W/kg, respectively [4]–[7]. Another important factor to be considered with EM signal absorption is the frequency. High frequency EM radiation with photon energy greater than 13.6 eV which is the ionization potential of hydrogen may lead to ionizing radiation. This may have a physiological effect that may lead to cancers. Examples of high frequency radiations include X-ray and gamma rays. Radiation of low frequencies such as radio and microwaves does not possess enough quantum energy to ionize the atom [8], and therefore, this was chosen to be the range of the EM study on human neurons. Certain frequency range from the RF EM may also alter the permeability of the blood brain barrier, causing structural damage to some enzymes of the plasma membrane. Researchers have also found that within the frequency range of 100 kHz and 6 GHz, restrictions on maximum average SAR should be specified in order to prevent heat stress and excessive localized temperature rise within the human body [9]. An analogous study of the MRI system led to the proper operating frequency of the study in order to avoid such issues. At MRI application, with a 1.5 T field, the gyromagnetic ratio of the hydrogen is 42.58 MHz/T. With 1.5 T, the resonance frequency will be nearly 64 MHz. At 3 T MRI coil strength, however, the resonance frequency will occur at nearly 128 MHz. Since the 64 MHz is approved for the MRI system, it is appropriate to consider this frequency for the study of the neuro research for the investigation of the impact of the EM and power intensity. The limits specified for the power and frequency standards will assist with the practical model intended for the future conduct towards the safety measures and guidance for health monitoring. The 64 MHz falls under these restrictions, and therefore, the power level will follow the safety guideline set by the US Government.

Engineers have known that there are tradeoffs that must accompany size changes when extrapolating EM results from their studies. The scaling up of a cell cultures or rodents, to a size many times larger than the original requires more than just the increase of the EM power. Rather, the studies must be redesigned if they are to perform the same functions throughout a size range spanning several orders of magnitude in body mass. Further, changes in size result in shifts in optimal or preferred frequencies of use due to tissue penetration, conductivity, permittivity, and other factors. In order to extrapolate these studies to humans we need to quantify the power absorption and its distribution. This absorbed energy is directly associated to the internal (inside the body) EM fields, not the incident (external) EM fields. They can be quite different, depending on the shape and size of the body, electrical properties, frequency, and the orientation with respect to the incident EM fields. Since every effect is associated to the internal fields, the cause and effect relationship must be formulated in terms of these fields. Internals fields of a mouse and a man exposed to the same external field can be drastically different, and consequently their biological response. On the other hand, different exposure conditions such as different frequencies may produce similar internal fields and therefore a similar biological effect.

## 2. Simulation Models

**Figure 1:**
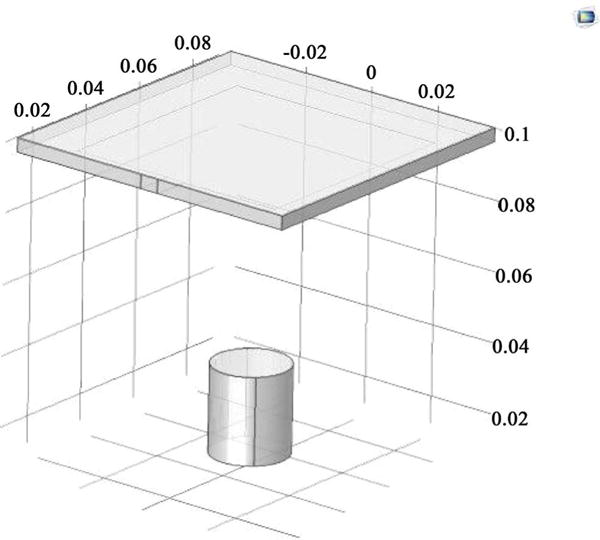
The model using in COMSOL for simulating. This Image shows the antenna used to generate the EM wave (Top) and the brain tissue sample (Bottom).

**Figure 2:**
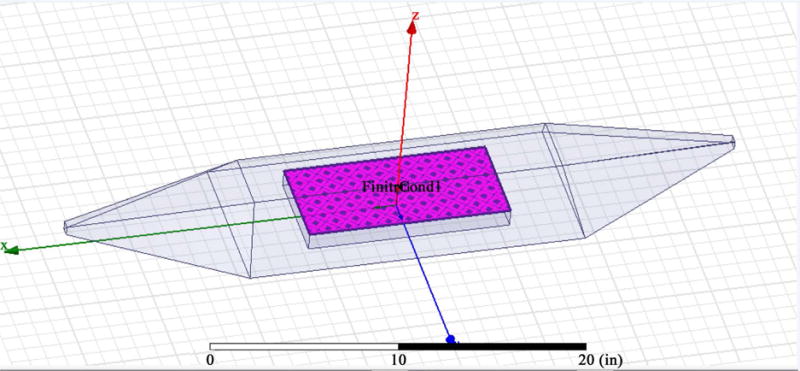
The EM HFSS model with 20 inch waveguide size with neurons in the middle.

The simulation conducted in this research was related to the solution of the combined EM and thermal diffusion equations. The antenna model from COMSOL with neuro cells modeled in cylindrical form is given in Figure 1. Figure 2 gives the HFSS model for the EM mesh simulation. The 20 inch wave guide with the field origin was placed in the center where the neuro cells reside.

### Mathematical Model

The heat equation used in the simulation is given by Equations (1) & (2) below:

(1) $$\rho C_{\rho }u\cdot \nabla dT+\nabla \cdot q=Q+Q_{\mathrm{bio}}$$

(2) $$Q_{\mathrm{bio}}=\rho_{\rho b}C_{\rho b}\omega_{b}(T_{b}-T)+Q_{\mathrm{met}}$$

where *Q_bio_* is the bio heat source, *q* = −*k*∇*T*, *T_b_* is the bio thermal temperature, and *ρ_ρb_* is the mass density of the material under bio thermal effect. The effect of the thermal takes place at the interface, hence it is important to consider the penetration depth throughout the materials. The thermal penetration depth is the distance that the heat may travel through.

The wave equations used in the simulation is given by Equations (3) & (4):

(3) $$\nabla \times u_{r}^{-1}(\nabla \times E)=k_{0}^{2}\left(\varepsilon_{r}-\frac{j\sigma }{\omega \varepsilon_{0}}\right)E$$

(4) $$n\times (\nabla \times (E))=\left(jk+\frac{1}{r}\right)n\times (E\times n)$$

where *σ* is the conductivity of the material, *ω* is the radian frequency, and *ε_r_* is the relative permittivity of the neuron cells. The boundary condition at the interface was given by matching the tangential components of the electric fields at the interface given by Equation (5):

(5) n×E=0

The SAR equation, which was estimated by the COMSOL software, is given by:

(6) $$E_{\mathrm{SAR}}=\sigma {\left[E\right]}^{2}/2\rho$$

## 3. The Proposed System

**Figure 3:**
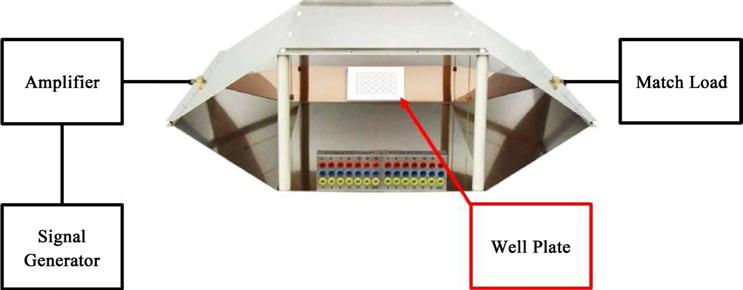
The model using in COMSOL for simulating.

**Figure 4:**
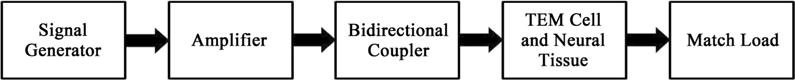
The flow chart showing the basic arrangement for the medical school’s test equipment.

Figure 3 describe the practical system which was considered for the simulation while Figure 4 provides the process of the various stages of the system.

In order to simulate the power absorption of the neurons at the target frequency, a simulation model was built. This model is designed to simulate the electric impedance of the neural tissue in a bulk state, meaning that a bulk set of material had the same electrical impedance of neural tissue that will be tested. Future work may include testing the power absorption experienced by individual neurons. The material properties used in the simulations are given in the appendix section.

### Human Neuronal Culture Model

**Figure 5:**
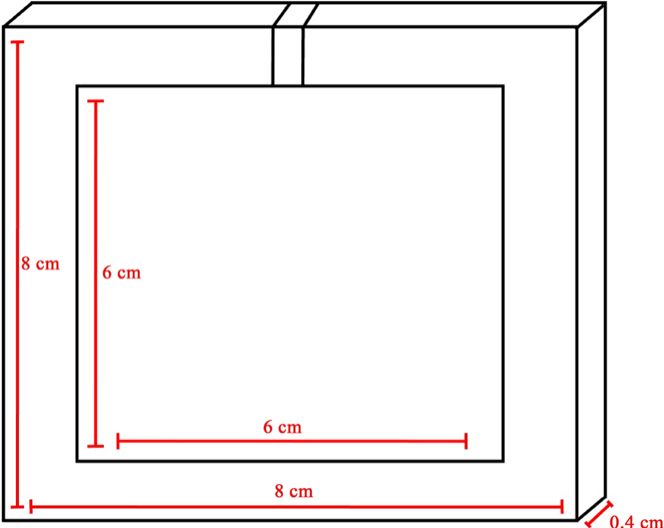
Patch antenna used in the COMSOL simulation with dimensions.

The Human Neuronal culture model takes into account two major assumptions for how the system works at full-scale. In the model, an antenna to serve as the source immediately next to the culture sample was used. Furthermore, in this model, the neuronal culture will respond as a bulk material rather than individual neurons. The culture sample is taken as a single well from the well plate where human neurons are residing, see diagram in Figure 3. The dimensions of the fetal brain culture sample cylinder used in this model are 1 cm radius and 2.5 cm height. The antenna type used for this simulation model was a patch antenna and is set to provide a radiation pattern that is directed towards the top of the brain culture sample. This is meant to demonstrate how the incident wave in the full-scale experiment could interact with the tissue samples. The dimensions of the patch antenna are shown in Figure 5.

## 4. Results and Discussions

The results obtained in the study using a transverse electromagnetic (TEM) cell that is an expanded coaxial transmission line operating in the TEM mode connected to the RF source through a coaxial cable may serve as a good practical model for small animal research setting. The parameters used in the simulation were appropriate, and the EM wave was transmitted throughout the waveguide region where the samples are, with minimum attenuation.

**Figure 6:**
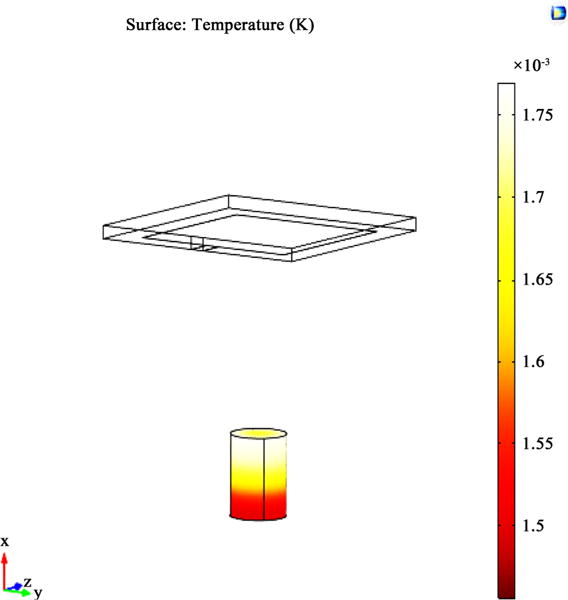
Simulation results from the COMSOL temperature study—determined at a frequency of 64 MHz.

The simulation results are shown in Figure 6. They provide the neural tissue response to the electromagnetic field for both temperature change and power absorbed, given in terms of SARs. The results from the COMSOL Bio heat transfer temperature study was taken when the brain tissue is exposed to 64 MHz EM wave. As expected, the majority of heating occurs at the top of the sample and becomes lower further through the sample. The temperature differential forms a gradient of heating throughout the sample, with the hotter regions (in white/yellow) towards the top moving to less heated regions (orange to ref) towards the bottom.

**Figure 7:**
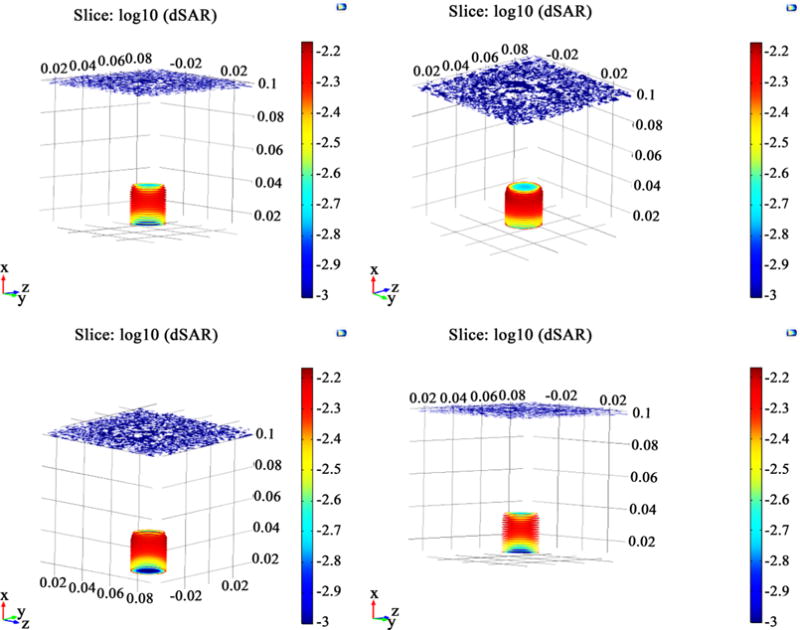
Simulation results from the COMSOL electromagnetic waves, frequency domain study showing the SAR results in segments-determine data frequency of 65 MHz.

The simulation results from the SAR electromagnetic study shows the results on a log scale. To visualize the results, the plots needed to be split into a number slices at different points within the sample. In order to best view these slices. The different views of the results show how the SAR value changes throughout the brain tissue sample. Here it can be seen that the largest degree of power absorbed (shown in red) occurs towards the top of the brain tissue sample. The penetration depth of the sample can be observed in Figure 7, the charge from the antenna source comes in at the top of the sample. The change then permeates outwards to the outside of the sample, as evidence by the blue region at the top of the sample and the red regions on the outside of the sample. Additionally, it can be observed that the majority of power absorbed by the material is concentrated towards the top of the outside regions of the sample. This can be seen by the deeper red region towards the top of the outside of the sample. These results match the temperature study in that the majority of the heating occurs in the regions the see the most power absorption from the SAR analysis.

**Figure 8:**
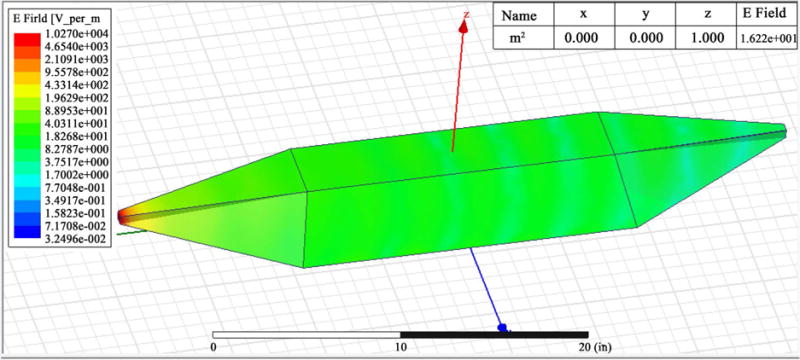
Electric field image captions at different time frames.

**Figure 9:**
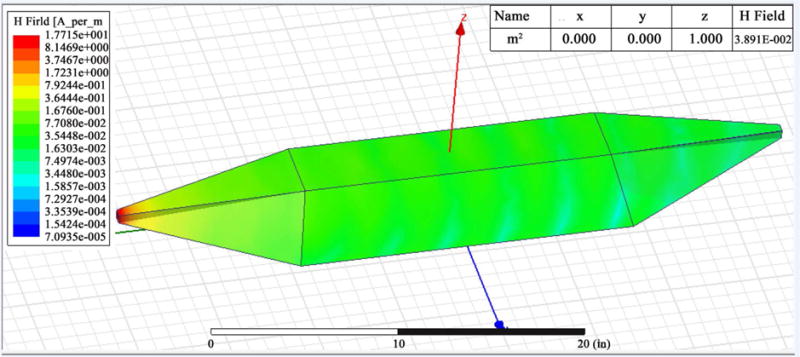
Magnetic field image frames at different time frames.

The EM simulations show E & H fields of less than 1% reflections (indicated by the blue color field), indicating a proper matching within the region where the samples are placed in the waveguide. The frame captures in Figure 8 gives complete cycle of E field and Figure 9 gives complete cycle of H field. The strongest E field is determined in order to achieve 0.6 W power in the middle of the waveguide. The maximum H field is estimated based on the wave impedance of the medium.

## 5. Conclusions and Future Considerations

The thermal and EM simulations presented in this work indicate promising data for a future practical model. Uniform magnetic and electric fields passing through the neuro samples with minimum reflections are important for the practical model. The proposed practical model has a capacity of fitting small animals for EM exposure. The ultimate goal of the project is to expand on the design of an antenna that can provide the research parameters discussed in this work. Temperature profile may set limitation factors for a given power density for absorption rate (SAR) applications. The maximum power strength for the maximum allowable temperature increase may also need to be estimated for practical considerations.

The 64 MHz used here with 0.6 W power applied to the neuro cells are appropriate to the human body application. This is analogous to the MRI operating frequency (for the 1.5 T machine). Other operating frequencies are not appropriate for this study. The study here will serve basis of activating the human neurons for Alzheimer disease purposes.

## Appendix: Parameters for the Model

**Table:**

Parameter Name	Value	Description
*cr-pcb*	5.23	Permittivity for the patch antenna board
*cr*0-*brain*	58.13	Permittivity for the brain tissue
*σ*0-*brain*	1.15 S/m	Conductivity for the brain tissue
*ρbrain*	1030 kg/m^3^	Density of brain tissue
*sdamping*	2.00E−4	Sampling parameter
*edamping*	4.00E−4	Sampling parameter
*soffset*	−1 S/m	Sampling parameter
*eoffset*	−50	Sampling parameter
*cblood*	3639 J/(kg*K)	Heat capacity of blood
*ρblood*	1000 kg/m^3^	Density of blood
*odamping*	1.08E−6 1/s	Sampling parameter
*ooffset*	7.80E−4 1/s	Sampling parameter
Frequency	64 MHz	Target frequency
